# A systematic analysis of the *in vitro* and *in vivo* functions of the HD-GYP domain proteins of *Vibrio cholerae*

**DOI:** 10.1186/s12866-014-0272-9

**Published:** 2014-10-25

**Authors:** Robert W McKee, Ankunda Kariisa, Benjamin Mudrak, Courtney Whitaker, Rita Tamayo

**Affiliations:** Department of Microbiology and Immunology, University of North Carolina at Chapel Hill, 125 Mason Farm Rd, CB# 7290 Chapel Hill, NC USA

**Keywords:** Second messenger, Cyclic diguanylate, Phosphodiesterase, Biofilm, Motility, Virulence, HD-GYP

## Abstract

**Background:**

The second messenger cyclic diguanylate (c-di-GMP) plays a central role in bacterial adaptation to extracellular stimuli, controlling processes such as motility, biofilm development, cell development and, in some pathogens, virulence. The intracellular level of c-di-GMP is controlled by the complementary activities of diguanylate cyclases containing a GGDEF domain and two classes of c-di-GMP phosphodiesterases containing an EAL or HD-GYP hydrolytic domain. Compared to the GGDEF and EAL domains, the functions of HD-GYP domain family proteins are poorly characterized. The human diarrheal pathogen *Vibrio cholerae* encodes nine putative HD-GYP domain proteins. To determine the contributions of HD-GYP domain proteins to c-di-GMP signaling in *V. cholerae*, we systematically analyzed the enzymatic functionality of each protein and their involvement in processes known to be regulated by c-di-GMP: motility, biofilm development and virulence.

**Results:**

Complementary *in vitro* and *in vivo* experiments showed that four HD-GYP domain proteins are active c-di-GMP phosphodiesterases: VC1295, VC1348, VCA0210 and VCA0681. Mutation of individual HD-GYP domain genes, as well as combinatorial mutations of multiple HD-GYP domain genes, had no effect on motility or biofilm formation of *V. cholerae* under the conditions tested. Furthermore, no single HD-GYP domain gene affected intestinal colonization by *V. cholerae* in an infant mouse model. However, inactivation of multiple HD-GYP domain genes, including the four encoding functional phosphodiesterases, significantly attenuated colonization.

**Conclusions:**

These results indicate that the HD-GYP family of c-di-GMP phosphodiesterases impacts signaling by this second messenger during infection. Altogether, this work greatly furthers the understanding of this important family of c-di-GMP metabolic enzymes and demonstrates a role for HD-GYP domain proteins in the virulence of *V. cholerae*.

**Electronic supplementary material:**

The online version of this article (doi:10.1186/s12866-014-0272-9) contains supplementary material, which is available to authorized users.

## Background

Cyclic diguanylate (c-di-GMP) is a bacterial second messenger first identified as an activator of cellulose synthesis in *Gluconacetobacter xylinus* [1]. Since its discovery, the number of processes known to be regulated by c-di-GMP in bacteria has expanded. c-di-GMP signaling has been shown to regulate numerous processes including, but not limited to, motility and biofilm production in numerous bacterial species (reviewed in [2-4]). In certain pathogens, c-di-GMP also influences virulence properties [5-17].

The concentration of c-di-GMP is controlled by the competing actions of two classes of enzymes: diguanylate cyclases, which are responsible for the synthesis of c-di-GMP from two molecules of GTP, and phosphodiesterases, which hydrolyze c-di-GMP forming two molecules of GMP. Diguanylate cyclase activity has been demonstrated in proteins containing GGDEF domains, and c-di-GMP phosphodiesterase activity has been observed in two unrelated protein domains, the EAL and HD-GYP domains [18-24]. EAL domain phosphodiesterases were the first to be described and have been more extensively studied in terms of structure and biochemical and biological function. Comparatively little is known about the functions of HD-GYP domain proteins.

The first protein containing an HD-GYP domain shown to act as a c-di-GMP phosphodiesterase was RpfG from plant pathogenic *Xanthomonas* spp*.* [21]. RpfG is a response regulator containing a phosphoreceiver (REC) domain and an HD-GYP domain. Along with the sensor histidine kinase RpfC, RpfG responds to extracellular diffusible signal factor (DSF), a cell-to-cell signaling factor. Evidence suggests that, in response to DSF, RpfC phosphorylates the REC domain of RpfG, triggering the phosphodiesterase activity of the HD-GYP domain [25,26]. The consequent decrease in intracellular c-di-GMP leads to derepression of Clp, a transcription factor inhibited by binding of c-di-GMP, activating transcription of genes necessary for virulence factor production [27-30]. Deletion of *rpfG* or amino acid substitutions in conserved residues of the HD-GYP domain, both of which abrogate c-di-GMP hydrolysis, resulted in decreased virulence factor secretion, and virulence factor secretion was restored in bacteria complemented with an EAL domain phosphodiesterase, indicating that c-di-GMP hydrolysis by RpfG is responsible for this phenotype [21,25,31,32].

In *Pseudomonas aeruginosa*, two genes encoding HD-GYP domains, PA4108 and PA4781, are necessary for full virulence in the Greater wax moth *Galleria mellonella* and for optimal swarming motility [33-35]. The HD-GYP phosphodiesterase PdeB of *Borrelia burgdorferi* plays a role in motility and contributes to survival of the bacterium in the tick vector *Ixodes scapularis* and to transmission of the bacterium to mice [13,36].

The genome of the human diarrheal pathogen *Vibrio cholerae* contains numerous genes encoding confirmed or putative c-di-GMP metabolic enzymes: 31 genes encoding GGDEF domains, 12 genes encoding EAL domains, 10 genes encoding tandem GGDEF-EAL genes, and 9 genes encoding HD-GYP domains [37,38]. A handful of diguanylate cyclases and EAL domain phosphodiesterase enzymes have been shown to impact motility, biofilm formation and virulence in animal models [14,15,39-46]. HD-GYP domain phosphodiesterases similarly have the potential to impact motility, biofilm formation and virulence of *V. cholerae* through modulation of c-di-GMP. However, relatively little is known about the function(s) of HD-GYP domain proteins in *V. cholerae*. The expression of four of these genes (VC2340, VCA0210, VCA0681, VCA0895) was upregulated in the presence of the quorum sensing autoinducers [47]. Ectopic over-expression of VCA0681 resulted in a reduced intracellular c-di-GMP concentration, indicating that VCA0681 possesses c-di-GMP phosphodiesterase activity [47]. Mutation of either VCA0681 or VCA0895 had no effect on biofilm formation in that study. Recently, bile acids, an extracellular signal encountered by *V. cholerae* in the intestine, were shown to activate and repress expression of the HD-GYP domain genes VC2497 and VC1295, respectively [48]. Furthermore, a VC1295 mutant has somewhat increased c-di-GMP and biofilm formation in the presence of bile acids, consistent with PDE function [48]. Beyond these experiments, not much is known about the function of HD-GYP containing proteins in *V. cholerae* or whether these proteins have phosphodiesterase activity.

Herein, we systematically analyze the biochemical and biological functions of the putative HD-GYP phosphodiesterases encoded by *V. cholerae.* We present *in vitro* and *in vivo* evidence that a subset of the HD-GYP domain proteins are enzymatically active and assess the roles of each HD-GYP domain gene in motility, biofilm formation and virulence of *V. cholerae.* This work greatly furthers the understanding of this important family of c-di-GMP metabolic enzymes and demonstrates a role for HD-GYP domain proteins in the virulence of *V. cholerae*.

## Methods

### Bacterial strains and growth conditions *V. cholerae*

C6706 and isogenic mutant strains (Additional file 1: Table S1) were cultured in Luria-Bertani (LB) broth containing 100 μg/ml streptomycin (Sm) at 37°C with aeration. *Escherichia coli* strains were grown in LB broth. When needed to select for plasmids, 50 μg/ml ampicillin (Amp) was added to the *V. cholerae* and *E. coli* cultures.

### DNA manipulations and strain construction

Primers used in the construction of in-frame deletions of genes encoding putative HD-GYP domain proteins are listed in Additional file 1: Table S3. Primers for deletion constructs are labeled with the locus tag of the target gene followed by F1, R1, F2 or R2 (e.g. VC1087F1 and VC2340R2). Deletions were made using standard allelic exchange methods [49]. Briefly, regions of homology upstream and downstream of the gene of interest were PCR amplified from *V. cholerae* C6706 genomic DNA using the geneF1+ geneR1 primers for the upstream fragment and geneF2 + geneR2 primers for the downstream fragment. The primers introduced restriction sites (Additional file 1: Table S3, underlined) that allowed cloning of the two PCR products to each other and into the suicide vector pCVD442. Ligation reaction products were transformed into DH5α λpir by electroporation, which were then selected by growth on LB-Amp agar. The desired clones were identified by screening by PCR with geneF1 + geneR2 primers and CVDseqF + CVDseqR primers that flank the multiple cloning site. Plasmids were purified from confirmed clones and transformed into Sm10 λpir cells by electroporation. Sm10 λpir strains containing the pCVD442 deletion constructs and *V. cholerae* recipient strains were grown on LB agar with Amp or Sm, respectively, then mated on LB agar lacking antibiotics for 6 hours at 37°C. Growth was collected from the agar plate and streaked onto LB-Sm-Amp agar to select for *V. cholerae* transconjugants. Single colonies were grown in 1 mL LB broth lacking antibiotics for 8 hours. Dilutions were plated onto LB-Sm agar containing 10% sucrose and incubated for 16 hours at 30°C. Sucrose-resistant, Amp-sensitive colonies were screened for deletion of the target gene by PCR using geneF0 and geneR2 primers.

We were unable to inactivate VC2497 by allelic exchange, so VC2497 was instead mutated by plasmid disruption with pGP704. An internal portion of VC2497 was amplified by PCR from *V. cholerae* C6706 genomic DNA using primers 2497koF and 2497koR. The product was digested with *Bgl*II and *Eco*RI and ligated into similarly digested pGP704. Ligation reaction products were transformed into DH5α λpir cells by electroporation, followed by selection on LB-Amp agar. Insert-positive clones were identified by PCR using 2497koF +2497koR. The resulting pGP704::‘VC2497’ plasmid was introduced into *V. cholerae* C6706 by a triparental mating with this recipient strain, DH5α λpir (pGP704::‘VC2497’), and *E. coli* (pRK2013::Tn9). The integration of pGP704 into the VC2497 locus was confirmed by PCR using 2497koF + CVDseqR.

For the “ΔHDGYP7” strain, the following genes were sequentially inactivated as above, in order: VCA0681, VCA0210, VC2340, VC1348, VCA0895, VC1295, and VCA0931.

### Construction of strains for overexpression

The genes encoding the 9 putative HD-GYP-domain containing proteins were amplified by PCR from *V. cholerae* C6706 using the geneF and geneHR primers listed in Additional file 1: Table S2 (e.g. VC1087F and VC1087HR primers were used to amplify VC1087). The exception is VC1295, which was amplified from *V. cholerae* N16961, because VC1295 from strain C6706 contains a mutation encoding a premature stop codon. The geneHR primers introduce a sequence encoding six histidine residues at the C-terminus of the translated protein product. The PCR products for each gene were digested with the restriction enzymes indicated in the table and ligated into pMMB67EH. The ligations were transformed into *E. coli* DH5α, with subsequent selection on LB-Amp agar. Clones were screened using primers geneF + geneHR and primers 67EHF and 67EHR, which flank the multiple cloning site of pMMB67EH. The correct plasmids were introduced into *E. coli* BL21 via electroporation.

To generate a construct for expressing an inactive allele of VC1348, we replaced the codons for the HD motif (amino acids 217 and 218) with two codons for alanine. Using C6706 genomic DNA as a template, regions upstream and downstream of (and including) the targeted sequence were amplified with primers VC1348F + VC1348R1-AA and VC1348F2-AA + VC1348HR, respectively. The two fragments, which overlap by 21 nucleotides including HD to AA mutation, were spliced together and the full product was amplified by PCR with primers VC1348F + VC1348HR. The resulting product was digested with *Sac*I and *Xba*I, ligated into similarly digested pMMB67EH, and transformed into *E. coli* DH5α. Correct clones were identified by PCR and sequencing, yielding pMMB67EH::VC1348AA-His6.

All expression plasmids were introduced into *V. cholerae* by conjugation.

### *In vitro* c-di-GMP phosphodiesterase assays

Purified His_6_-WspR protein from *Pseudomonas aeruginosa*, which contains a catalytically active GGDEF domain, was used to synthesize the radiolabelled c-di-GMP substrate for the phosphodiesterase enzymatic assays [50]. Purified His_6_ –WspR (10 μL) was incubated overnight at 37°C with 5 μL of [α-^32^P]GTP (3000 Ci/mmol; PerkinElmer Life Sciences) and 5 μL of 100 mM GTP buffer containing 75 mM Tris, pH 8, 250 mM NaCl, 25 mM KCl, and 10 mM MgCl_2_. Subsequent steps were carried out as described [24].

*E. coli* BL21 containing either HD-GYP overexpression plasmid or vector were grown in LB-Amp broth overnight at 37°C with aeration, then diluted 1:100 in 12 mL of LB-Amp containing 1 mM IPTG. Cultures were grown at 30°C until an OD_600_ of 0.6-0.7 was reached. Cells were collected by centrifugation at 5000 × g for 10 minutes at 4°C. The cells were suspended in 1 mL of phosphodiesterase buffer containing 75 mM Tris, pH 8, 25 mM KCl, 25 mM MnCl_2_, and 10% glycerol. The cells were lysed by three rounds of freeze-thaw followed by sonication. Samples were centrifuged at 5,000 × g for 10 minutes at 4°C. Supernatants (soluble material) were removed and stored at –20°C prior to use in phosphodiesterase enzymatic assays. Reaction products were identified based on previously determined migration in KH_2_PO_4_ buffer [51].

Soluble fractions from *E. coli* BL21 overexpressing HD-GYP genes were tested for phosphodiesterase activity by incubating 18 μL of cell lysates with 2 μL of [^32^P]c-di-GMP for 0 min to 30 min at room temperature as previously described [14]. As negative controls, cell lysates from similarly treated *E. coli* with empty vector and a buffer-only control were tested. The reactions were stopped by spotting 0.5 μL of the reactions on PEI-cellulose and allowing the spots to air-dry. Nucleotides were separated by thin layer chromatography in 1.5 M KH_2_PO_4_, pH 3.65. PEI-cellulose plates were air-dried. Reaction products were visualized by phosphorimagery using a Storm Phosphorimager (GE Healthcare).

### Phenotype assays

Biofilm production was assayed by crystal violet staining as described previously [52,53]. Briefly, biofilms of *V. cholerae* were grown statically in LB broth in 13 mm diameter glass culture tubes at room temperature (~23°C) for 24 hours. Where indicated, 1 mM IPTG was added to induce phosphodiesterase gene expression. Unattached cells were removed, and the remaining biofilms were rinsed and stained with 0.1% crystal violet. The stained biofilms were washed with water, and then the stain was solubilized with 2 ml of 50% v/v ethanol. The biofilm material was dispersed using a sonicating water bath. Biofilm formation was determined by measuring the absorbance at 540 nm. At least 3 independent samples were assayed.

Motility was measured using soft agar assays as described [54]. Strains were grown overnight at 37°C on LB +1.5% agar. Single colonies were inoculated into motility plates (1% tryptone, 0.5% NaCl, 0.3% agar). Where indicated, 1 mM IPTG was added the plates to induce phosphodiesterase gene expression. Motility plates were incubated for 16 hours at 30°C, and colony swarm diameters were measured. Three independent experiments of at least 4 replicates of each strain were performed.

### Western blots

BL21 and *V. cholerae* strains containing HD-GYP expression vectors were grown in LB-Amp broth overnight at 37°C with aeration, then diluted 1:100 in LB-Amp containing 1 mM IPTG. Cultures were grown at 30°C with aeration to an OD_600_ of ~0.7. Cells were collected by centrifugation at 12,000 × *g* and suspended in 100 μL of 2 × Laemmli sample buffer [55]. Samples were separated by electrophoresis on a 12% polyacrylamide SDS gel then transferred to a nitrocellulose membrane. Western blots were carried out according to standard procedures. Primary mouse His-6 antibody (ThermoScientific) was used at a 1:1000 dilution. Dylight 800 goat α-mouse secondary antibody (ThermoScientific) was used at a 1:15000 dilution. Blots were imaged using an Odyssey imager (LI-COR Biosciences).

### *In vivo* competition experiments

The animal experiments were done in accordance with protocol 12-239 approved by the Institutional Animal Care and Use Committee at the University of North Carolina at Chapel Hill. Mouse *in vivo* competition experiments were carried out in 5 day old CD-1 mice as described previously [56]. Briefly, HD-GYP mutant strains were mixed 1:1 with WT ∆*lacZ* mice in 0.85% NaCl. To determine the ratio of mutant to WT in the inocula, these mixtures were plated on LB-Sm agar containing 40 μg/mL 5-bromo-4-chloro-3-indolyl-β-D-galactopyranoside (X-Gal), which allows differentiation of mutant and Δ*lacZ* bacteria (blue versus white colonies, respectively). Mice were inoculated intragastrically with ~10^5^ total bacteria. Animals were sacrificed 21 hours post-inoculation. Small intestines were harvested and homogenized, then dilutions were plated on LB-Sm agar containing X-Gal. The competition index was determined by dividing the ratio of mutant/Δ*lacZ* bacteria in the homogenates (output) by the ratio of mutant/Δ*lacZ* bacteria in the inocula. In parallel, the mixtures used to inoculate mice for *in vivo* competition experiments were also diluted 1:1,000 into 2 ml of fresh LB broth and incubated overnight to determine the *in vitro* competition index. At least 5 mice were used for each competition. Data were analyzed by the Wilcoxon signed-rank test, with values compared to a hypothetical value of 1.

## Results

### A subset of the 9 genes of *V. cholerae* is predicted to encode enzymatically active HD-GYP domains

*V. cholerae* N16961 encodes 9 putative HD-GYP domain c-di-GMP phosphodiesterases with variable lengths, domain architectures and predicted cellular localizations. Most of the proteins are estimated to be approximately 400-500 amino acids in length, though VCA0895 is considerably larger, with 981 amino acids. Two HD-GYP domain proteins, VC1295 and VCA0895, have predicted transmembrane domains and are predicted to be localized in the inner membrane. As is common of c-di-GMP metabolic enzymes, all 9 HD-GYP domain proteins contain additional domains potentially involved in regulating protein function. Three HD-GYP domain proteins, VC1087, VC1348 and VCA0210, possess a phosphoreceiver (REC) domain, suggesting that they may be modulated by phosphorylation. Two of the proteins, VC1295 and VCA0895, contain a domain present in Histidine kinases, Adenylate cyclases, Methyl-accepting proteins and Phosphatases (HAMP), which is a linker domain suggested to function in intramolecular signal transduction [57-59]. Three contain additional domains of unknown function: VC2497, DUF3391; VCA0210, DUF3369; and VCA0931, DUF3392. Interestingly, VCA0681 contains tandem HD and HD-GYP domains. Together, these 9 putative c-di-GMP phosphodiesterases have the potential to respond to myriad signals to modulate intracellular c-di-GMP.

**Figure 1:**
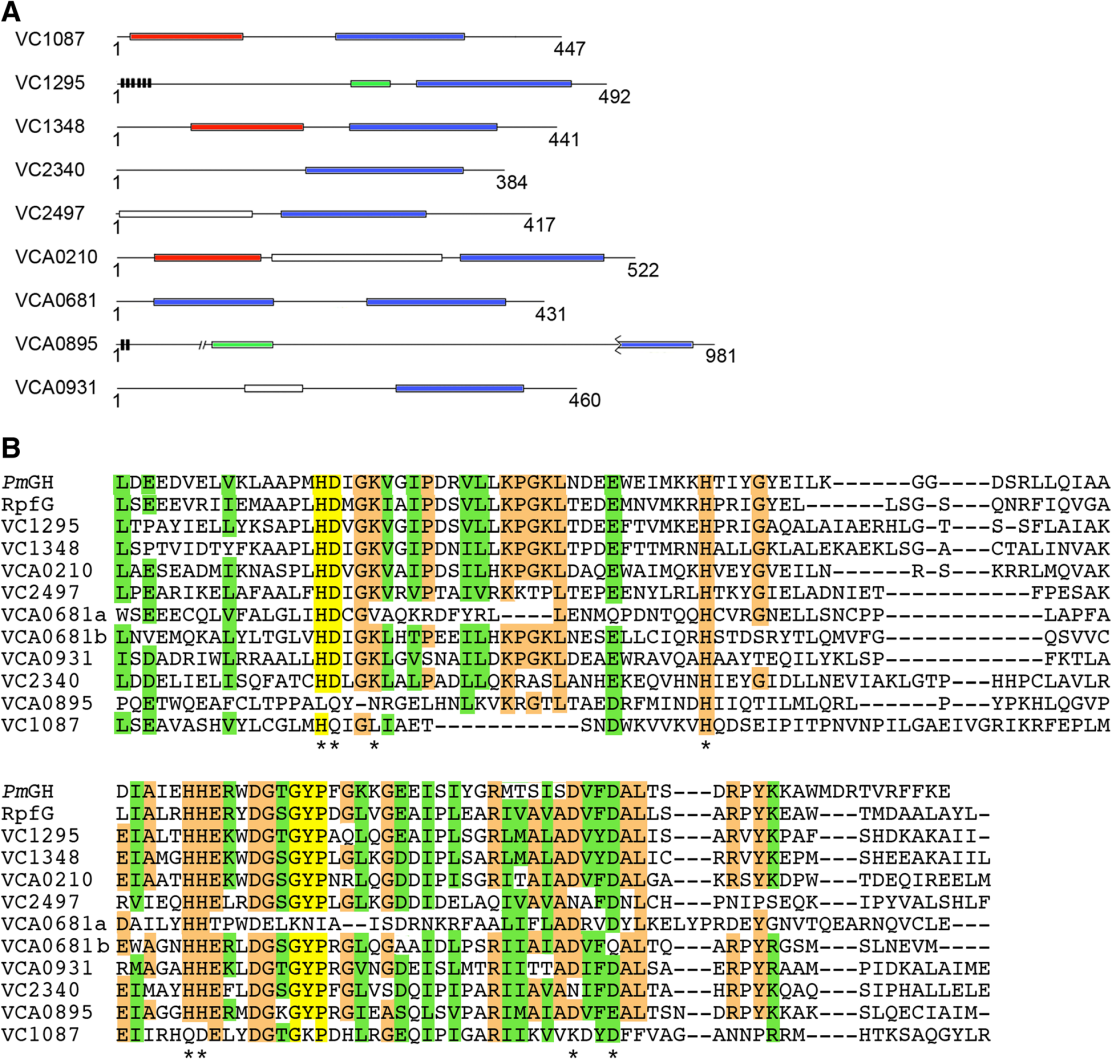
**Domain architectures and sequence conservation of putative HD-GYP domain proteins of** ***V. cholerae*** **. (A)** Predicted domains of the nine HD-GYP domain proteins encoded by *V. cholerae*. Shown are HD-GYP domains (blue), phosphoreceiver domains (red), HAMP domains (green), domains of unknown function (white) and putative transmembrane domains (black). The lengths of the proteins (number of amino acids) are indicated. **(B)** Alignment of *V. cholerae* HD-GYP domains. The HD-GYP domain amino acid sequences from *V. cholerae*, including the two tandem domains in VCA0681, were aligned to the HD-GYP domains of RpfG from *X. campestris* and *Pm*GH from *Persephonella marina* using Clustal Omega software. Highlighted are the HD and GYP motifs (yellow), identical amino acid residues (orange) and conserved amino acid residues (green). Asterisks indicate residues previously shown to be required for enzymatic activity of *Pm*GH [61].

Previous studies have used genetic and biochemical approaches to identify the residues of HD-GYP domain proteins that are important for catalytic activity [21,60-62]. Mutational analysis of *Pm*GH, an HY-GYP domain phosphodiesterase from *Persephonella marina* for which the c-di-GMP cocrystal structure has been solved, identified at least 11 amino acids required for full phosphodiesterase activity [60]. Based on conservation of residues deemed critical for activity of the HD-GYP domain phosphodiesterase *Pm*GH, four of the 9 putative HD-GYP domain proteins encoded by *V. cholerae* are predicted to be enzymatically active: VC1295, VC1348, VCA0210 and VCA0931 (Figure 1B). VC2497, VC2340 and VCA0681 (predicted to have tandem HD and HD-GYP domains, labeled “a” and “b” in Figure 1, respectively) contain one non-conserved residue required for activity of *Pm*GH. Two, VC1087 and VCA0895, lack an intact HD motif and are expected to be enzymatically inactive.

### Four HD-GYP domain proteins of *V. cholerae* are enzymatically active *in vivo*

**Figure 2:**
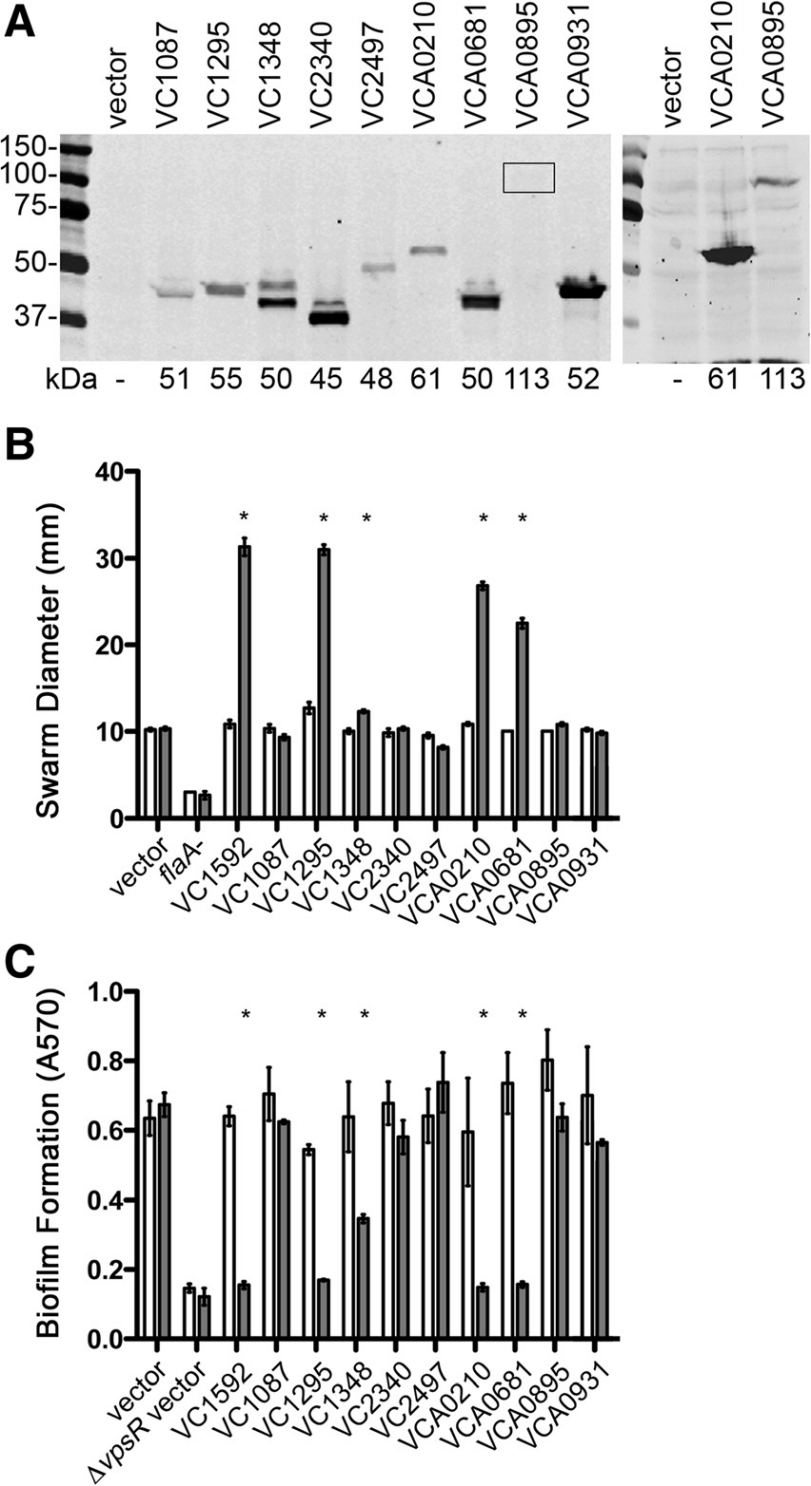
**Ectopic over-expression of HD-GYP genes in** ***V. cholerae.*** Each gene encoding a putative HD-GYP domain protein, tagged with a 6-histidine sequence at the 3′ end, was expressed ectopically in *V. cholerae* using an IPTG-inducible promoter. **(A)** Production of each protein in *V. cholerae* after ~3 hours growth in the presence of 1 mM IPTG was assessed by western blot using anti-His6 antibodies. The predicted molecular weights of each protein, in kDa, are indicated at the bottom. Right: Expression of the large, predicted membrane protein VCA0895 was relatively poor; detection of the recombinant protein required loading more sample. **(B)** Motility of *V. cholerae* expressing each of the HD-GYP domain genes was assayed in soft agar medium as an indication of c-di-GMP hydrolysis, which augments flagellar motility of *V. cholerae*. Each strain was inoculated into motility medium with (grey bars) or without (white bars) 0.5 mM IPTG. The diameter of each motility swarm was measured after 20 hours incubation at room temperature. Shown are means and standard deviations for three biological replicates. **(C)** Biofilm formation by *V. cholerae* expressing each of the HD-GYP domain encoding genes was assessed as an indication of c-di-GMP hydrolysis, which decreases biofilm formation by *V. cholerae*. Each strain was grown in LB broth with (grey bars) or without (white bars) 0.5 mM IPTG to induce gene expression. Biofilm was assayed using standard crystal violet methods. Shown are means and standard deviations for three biological replicates. *P <0.05 by two-way ANOVA with Bonferroni’s post-test.

To assess the enzymatic activity of each putative HD-GYP domain phosphodiesterase, we used motility and biofilm formation, processes well-known to be inhibited and activated by c-di-GMP in *V. cholerae*, respectively, as phenotypic indicators of *in vivo* enzymatic activity [14,40,43,63]. Each HD-GYP domain-encoding gene was ectopically expressed in *V. cholerae* under the regulation of an inducible promoter. We used alleles containing a C-terminal sequence encoding a 6-histidine tag, allowing us to confirm expression by western blot. Each strain produced a His6-tagged protein of approximately the anticipated molecular weight, though the abundance of the proteins varied (Figure 2A, left). Expression of VCA0895-His6 was relatively poor; visualization required loading twice the amount of sample and a longer exposure of the membrane (Figure 2A, right).

The strains were then assayed for altered motility and biofilm formation compared to the parental strain carrying vector only, each with and without IPTG to induce gene expression. *V. cholerae* expressing the characterized EAL domain phosphodiesterase VC1592 was included as a positive control for both experiments [46]. Expression of VC1592, VC1295, VC1348, VCA0210, and VCA0681 significantly increased motility of *V. cholerae* through 0.3% agar medium, as evinced by expanded diameters of motility (Figure 2B). Expression of the same genes significantly decreased biofilm formation in rich medium on glass (Figure 2C). These results are consistent with reduced intracellular c-di-GMP upon expression of c-di-GMP phosphodiesterases and suggest that VC1295, VC1348, VCA0210 and VCA0681 encode active HD-GYP domain c-di-GMP phosphodiesterases. As expected, VC1087 and VCA0895, which lack an intact HD motif, had no effects on motility or biofilm formation.

### A subset of HD-GYP domain proteins of *V. cholerae* are enzymatically active *in vitro*

**Figure 3:**
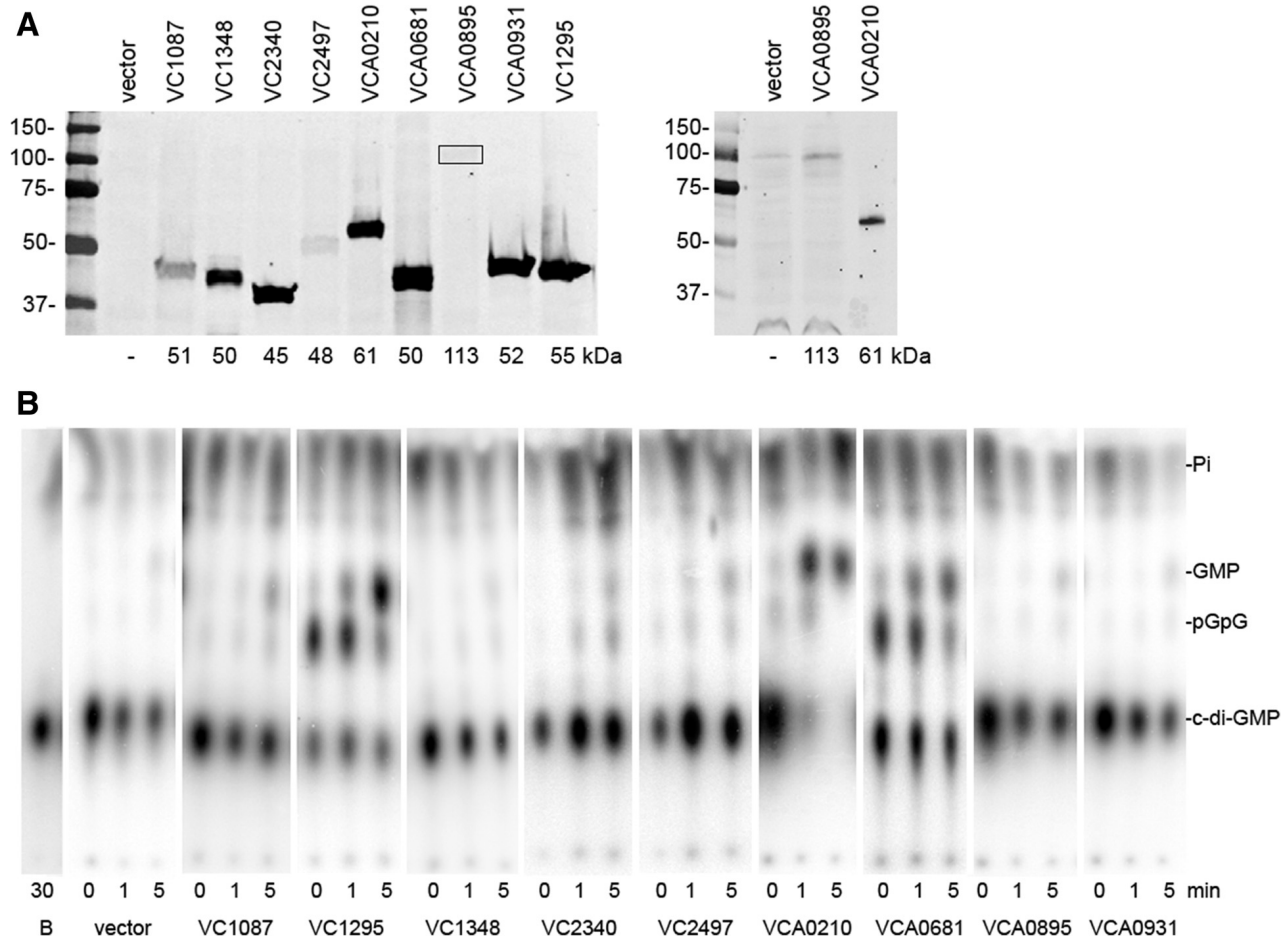
**Heterologous expression of HD-GYP genes in** ***E. coli*** **.** Each gene encoding a putative HD-GYP domain protein, tagged with a 6-histidine sequence at the 3′ end, was expressed ectopically in *E. coli* BL21. **(A)** Production of each protein after ~3 hours growth in the presence of 1 mM IPTG was assessed by western blot using anti-His6 antibodies. The predicted molecular weights of each protein, in kDa, are indicated at the bottom. Right: Expression of the large, predicted membrane protein VCA0895 was relatively poor; detection of the recombinant protein required loading more sample. **(B)** Lysates of *E. coli* BL21 expressing each of the HD-GYP domain-encoding genes were tested for the ability to hydrolyze c-di-GMP as described in the Methods. Buffer only (“B”) and lysates from *E. coli* BL21 with vector only were included as negative controls. The radiolabeled c-di-GMP substrate has a relative mobility (R_f_) of ~0.3 and appears for all samples at t =0, when the reaction was initiated by addition of [^32^P]-c-di-GMP. The GMP reaction product appears at R_f_ ~0.65.

Because the HD-GYP domain proteins could affect biofilm formation and swimming motility independently of c-di-GMP hydrolytic activity, we also assayed their enzymatic activity *in vitro*. The same plasmids used for expression in *V. cholerae* were transformed into *E. coli* BL21. These strains were grown in the presence of IPTG to induce gene expression; *E. coli* BL21 with vector only was included as a negative control. Production of His6-tagged HD-GYP domain proteins was confirmed by western blot (Figure 3A, left). As in *V. cholerae*, *E. coli* expressing VCA0895 produced less protein, and loading 2-fold more sample was needed to detect VCA0895-His6 (Figure 3A, right). Culture lysates were tested for the ability to hydrolyze c-di-GMP by incubating them with radiolabeled c-di-GMP. The reaction products were separated by TLC and visualized by phosphorimagery. *In vitro*, VC1295, VCA0210 and VCA0681 reproducibly hydrolyzed c-di-GMP (R_f_ ~0.3) at levels above that of the vector-only negative control. Notably, in these reactions, both linearized pGpG (R_f_ ~0.55) and GMP (R_f_ ~0.65) were apparent. VC1348, which showed activity *in vivo*, failed to hydrolyze c-di-GMP above background levels. VC1087 and VCA0895, as expected, were inactive.

### The effect of VC1348 expression on swimming motility require(s) c-di-GMP phosphodiesterase activity

**Figure 4:**
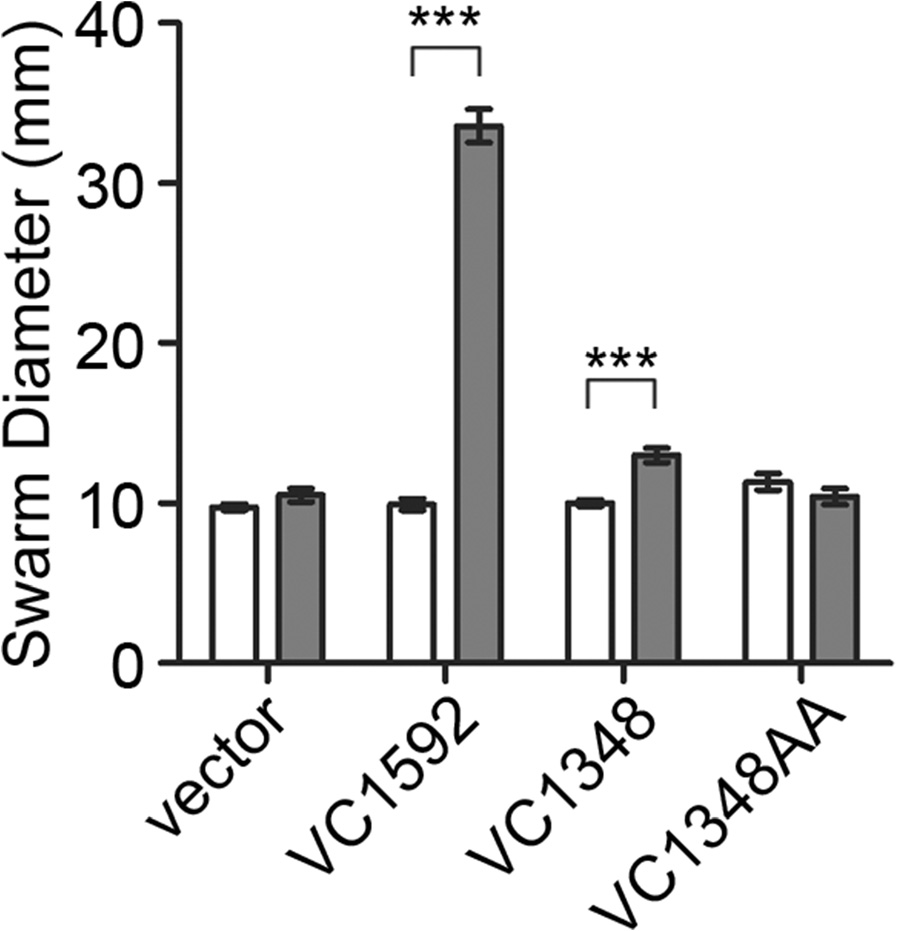
**The HD residues of VC1348 are required for** ***in vivo*** **function.** The genes encoding wild type VC1348 and VC1348 with alanine substitutions in the HD motif (VC1348AA) were expressed ectopically in *V. cholerae* using an IPTG-inducible promoter. The EAL domain PDE VC1592 was included as a positive control. The motility of *V. cholerae* bearing expression plasmids or vector control was assayed in soft agar medium as an indication of c-di-GMP hydrolysis, which augments flagellar motility of *V. cholerae*. Each strain was inoculated into motility medium with (grey bars) or without (white bars) 0.5 mM IPTG. The diameter of each motility swarm was measured after 20 hours incubation at room temperature. Shown are the means and standard deviations for six biological replicates. ***P <0.001 by two-way ANOVA with Bonferroni’s post-test.

For eight of the nine HD-GYP domain proteins, the *in vivo* over-expression and *in vitro* enzymatic assays were in agreement, indicating that VC1295, VCA0210 and VCA0681 are functional c-di-GMP phosphodiesterases. Expression of the remaining HD-GYP domain protein, VC1348, increased motility and decreased biofilm formation of *V. cholerae*, consistent with reduced c-di-GMP in this strain. However, no c-di-GMP phosphodiesterase activity could be detected *in vitro*. To determine if the phenotypic changes upon VC1348 expression in *V. cholerae* were due to phosphodiesterase activity, the effect of expressing a mutant allele of VC1348 on *V. cholerae* motility was assessed as described above. The resulting mutant protein, herein named VC1348AA, contains alanine substitutions in the HD motif (amino acids 217 and 218), which has been shown to be required for c-di-GMP phosphodiesterase activity [21]. Expression of the positive control gene VC1592 and wild type VC1348 significantly increased motility of *V. cholerae* through 0.3% agar, while expression of VC1348AA did not (Figure 4). These results indicate that the HD motif of VC1348, and therefore c-di-GMP hydrolysis by this protein, is required for function *in vivo*.

### Expression of the HD-GYP domain genes in *V. cholerae* during planktonic and biofilm growth

**Figure 5:**
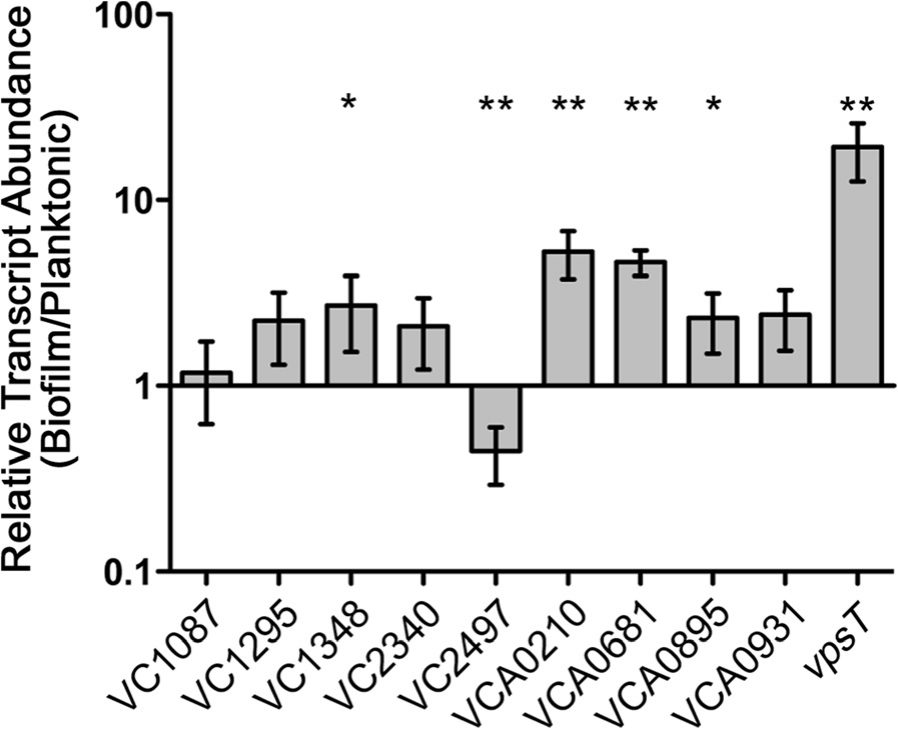
**Abundance of mRNA encoding HD-GYP domain proteins in** ***V. Cholerae*** **biofilms.** qRT-PCR was used to measure the transcript abundance for each HD-GYP mRNA, the reference housekeeping gene *rpoB*, and the biofilm-induced control gene *vpsT*. The relative differences in transcript abundance between biofilm and planktonic *V. cholerae* cells were determined as described in the Methods. *P <0.05 by unpaired *t*-test comparing biofilm to planktonic values, indicating a significant change in transcript abundance in biofilm relative to planktonic cells.

We postulated that the expression of the HD-GYP domain-encoding genes may be differentially expressed during growth of *V. cholerae* in a biofilm compared to in planktonic culture. For instance, one or more of the genes might be down-regulated, leading to increased intracellular c-di-GMP and enhanced biofilm formation. To get a picture of the transcriptional profiles of the HD-GYP domain genes, we used qRT-PCR to determine the relative expression of each HD-GYP domain gene in biofilm and planktonic culture. The *vpsT* gene, previously determined to be upregulated in a biofilm, was included as a positive control [64]. Several transcripts, VC1348, VCA0210, VCA0681, and VCA0895, were significantly more abundant in biofilm cells than in planktonic cells (Figure 5). Only one, VC2497, was significantly reduced in biofilms. These data suggest that c-di-GMP phosphodiesterases can be upregulated in biofilms, for example to counter the activities of diguanylate cyclases during biofilm development.

### Analysis of the roles of HD-GYP domain phosphodiesterases in motility, biofilm and virulence of *V. cholerae*

**Figure 6:**
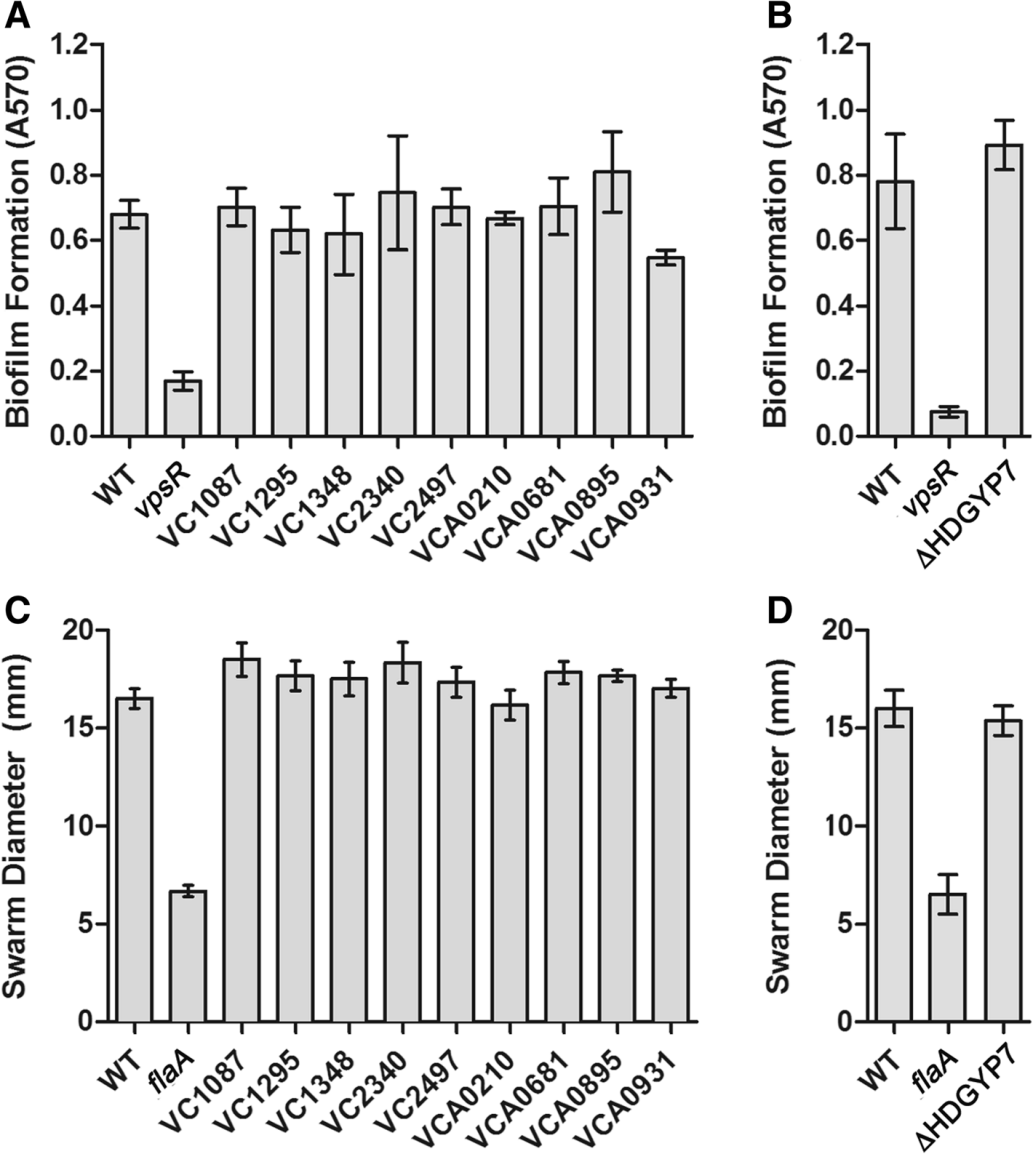
**Biofilm and motility phenotypes of** ***V. cholerae*** **strains containing mutations in one or more HD-GYP domain encoding genes.** *V. cholerae* containing mutations in single **(A)** or multiple **(B)** HD-GYP genes were assayed for biofilm formation after 48 hours static growth in rich medium using crystal violet staining. A *vpsR* mutant was included as a negative control. Shown are the means and standard deviations for three biological replicates. *V. cholerae* containing mutations in single **(C)** or multiple **(D)** HD-GYP genes were assayed for motility in soft agar medium. A *flaA* mutant was included as a negative control. Swarm diameters were measured after 20 hours growth at room temperature. Shown are the means and standard deviations for three biological replicates.

To assess the functions of the HD-GYP domain phosphodiesterases in *V. cholerae*, in-frame deletions were made in each gene, and the mutants were tested for altered biofilm formation and motility. None of the mutants showed phenotypes different from the WT parental strain (Figure 6A and C). To address the possibility that two or more of the HD-GYP proteins have redundant functions, additional mutants were made in which multiple HD-GYP encoding genes were deleted. None of these mutants, including strain ΔHDGYP7 (∆VCA0681 ∆VCA0210 ∆VC2340 ∆VC1348 ∆VCA0895 ∆VC1295 and ∆VCA0931), which contains mutations in all HD-GYP genes encoding functional enzymes, were phenotypically different from the WT parent (Figure 6B and D).

**Figure 7:**
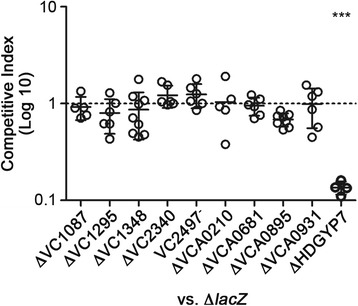
**Assessment of virulence phenotypes** ***V. cholerae*** **HD-GYP gene mutants using the infant mouse model.** Competition experiments were performed using the infant mouse model. Each mutant was co- inoculated in equal numbers with *V. cholerae* Δ*lacZ* (1:1 ratio) into 5-day-old CD1 mice. The bacteria present in the small intestine after 21 hours were enumerated on LB agar plates containing X-gal to differentiate mutant bacteria (blue colonies) from Δ*lacZ* bacteria (white colonies). The competition index (CI) was calculated as [(mutant CFU/Δ*lacZ* CFU)output]/[(mutant CFU/Δ*lacZ* CFU)input]. Each circle represents the CI from an individual animal, and the bars indicate the median. At least 4 mice were used per competition. ***P <0.001, one sample *t*-test comparing values to a hypothetical value of 1 (i.e., no difference between mutant and wild-type) and by one-way ANOVA.

Previous work with the infant mouse model has shown that c-di-GMP inhibits the ability of *V. cholerae* to effectively colonize the intestine. Mutation of the EAL domain phosphodiesterase VieA reduced fitness of *V. cholerae* O395 in the infant mouse [15]. We thus tested each of the HD-GYP mutants for colonization of the infant mouse small intestine using a competition assay. In this study, equivalent numbers of differentially labeled WT (*lacZ*+) and mutant (Δ*lacZ*) bacteria were co-inoculated into infant mice. After 21 hours, the small intestines were harvested, homogenized and plated on media containing X-gal to enumerate the WT and mutant bacteria. Competition indices were determined by dividing the ratio of mutant: WT bacteria in the tissues to the ratio of mutant: WT bacteria in the input mixtures. None of the individual HD-GYP gene mutations affected the ability of *V. cholerae* to colonize the infant mouse small intestine (Figure 7). Interestingly, the additive mutation of 7 HD-GYP domain genes (ΔHD-GYP7), including all those that are enzymatically active PDEs, led to a significant decrease in bacterial burden in the small intestine (mean CI = 0.135, *P* <0.001). This may be attributable in part to overall reduced fitness of the mutant, as the ΔHD-GYP7 strain was slightly attenuated for growth in LB compared to the Δ*lacZ* control (mean CI = 0.639, *P* <0.05). These results indicate that two or more HD-GYP genes in combination are important for virulence of *V. cholerae*. Alternatively, the cumulative effects of multiple PDE on intracellular c-di-GMP levels during infection may impair host colonization.

## Discussion

The c-di-GMP signaling system of *V. cholerae* is complex, including a large number of c-di-GMP synthases, hydrolases, effectors and regulatory targets. This work presents a methodical analysis of the functions of all 9 HD-GYP domain proteins encoded by *V. cholerae*.

Using *in vitro* and *in vivo* approaches, we found that a subset of the HD-GYP domain proteins possesses c-di-GMP phosphodiesterase activity. Activity correlates well with conservation of residues previously determined to be required for activity and with the overall consensus sequence of the HD-GYP domain [61,65]. We expected that VC1295, VC1348, VCA0210 and VCA0931 would be enzymatically active proteins; each contains conserved residues previously shown to be required for activity of *Pm*GH [61]. In addition, each contains conserved residues in the GYP motif region and other residues involved in recognition of c-di-GMP [61,66]. We consistently observed *in vivo* and *in vitro* activity for VC1295 and VCA0210. VC1348 caused increased motility and reduced biofilm formation when expressed in *V. cholerae*, suggesting that it is able to hydrolyze c-di-GMP *in vivo*, but we did not detect enzymatic activity *in vitro*. Mutating the HD residues required for phosphodiesterase activity of HD-GYP domain proteins ablated the effect of VC1348 expression on motility of *V. cholerae*. These results indicate that VC1348 is indeed a functional c-di-GMP phosphodiesterase in *V. cholerae*. We suspect that expression of VC1348 in *V. cholerae* allows VC1348 to be post-translationally activated, likely though its REC domain, but that the activating partner is absent in *E. coli* BL21. The adjacent, divergently transcribed gene VC1349 encodes a putative sensor kinase/response regulator and may regulate VC1348 in *V. cholerae*. VCA0931 failed to demonstrate c-di-GMP hydrolysis, neither affecting biofilm formation or motility .19pt]?>of *V. cholerae* nor hydrolyzing c-di-GMP *in vitro*. VCA0931 possesses an N-terminal domain of unknown function, which may post-translationally regulate the HD-GYP domain in response to specific cues lacking in our assays, or mask activity of the HD-GYP domain [33].

As we anticipated, VC1087 and VCA0895, which lack the HD dyad characteristic of HD metal-dependent phosphohydrolases, showed no evidence of c-di-GMP hydrolytic activity *in vivo* and *in vitro*. The activities of a subset of the HD-GYP domain proteins could not be easily predicted due to lack of conservation of one or more residues expected to be required for activity: VC2497, VC2340 and VCA0681 [21,61]. While VC2497 and VC2340 were inactive in all assays used, VCA0681 consistently showed phosphodiesterase activity. VCA0681 is one of the few HD-GYP proteins studied to date. Its enzymatic activity was previously demonstrated and its crystal structure was solved [47,60]. The differences between the inactive VC2497 and VC2340 and the active HD-GYP domain proteins that determine enzymatic functionality are not readily apparent. Indeed, VCA0681 is a functional PDE despite lacking an aspartic acid required for activity of *Pm*GH (D308 in PmGH, Q373 in VCA0681). The substitution of an aspartic acid with an asparagine in VCA0681 may preserve enzymatic activity, or the requirements of PmGH may not be universal to all HD-GYP domain PDEs. Further characterization of structure-function relationships of HD-GYP domain phosphodiesterases from a variety of organisms will be necessary to define the features required for activity.

Analysis of the expression of the HD-GYP domain genes in *V. cholerae* showed that VC1348, VCA0210, VCA0681 and VCA0895 transcripts are significantly higher in biofilm-derived *V. cholerae* than in planktonic cells. Interestingly, three of these genes, VCA0210, VCA0681 and VCA0895 were among the four HD-GYP domain genes regulated by quorum sensing [47]. Thus, high cell density in the *V. cholerae* biofilm may be the signal controlling expression of these genes.

We found that mutation of individual HD-GYP domain genes did not affect c-di-GMP regulated phenotypes of *V. cholerae* under the conditions tested. Even removal of all active (and many inactive) HD-GYP domain proteins in a single strain had no effect on motility or biofilm production. There are several possible reasons for the lack of phenotypic differences from the parent strain. First, it remains possible that HD-GYP domain phosphodiesterases, individually or as a class, impact c-di-GMP regulated processes other than those investigated here. Second, not all HD-GYP domain proteins are likely to be produced or enzymatically active under the conditions tested, so eliminating them may not have an observable effect. Our qRT-PCR studies of HD-GYP gene expression in planktonic and biofilm cultures support this possibility—expression of each HD-GYP domain gene is variable within those conditions. The observation that a VC1295 mutant produces somewhat more biofilm in the presence of bile acids further indicate that specific extracellular cues may be necessary to stimulate HD-GYP production and/or activity [48].

There may also be strain-dependent differences in the panel of c-di-GMP metabolic enzymes used, and different results might be obtained using a different *V. cholerae* strain. For example, mutation of the EAL domain phosphodiesterase VieA in the classical biotype of *V. cholerae* (strain O395) causes marked effects on motility, biofilm formation and virulence gene expression [15,40]; the same mutation in the El Tor strain C6709 has no effect on these processes [14,47].

HD-GYP domain proteins can also perform their regulatory function via physical interactions with other proteins, independently of c-di-GMP phosphodiesterase activity. In *Xanthomonas spp.*, for example, the HD-GYP phosphodiesterase RpfG interacts with two diguanylate cyclases to regulate motility [21,66,67]. The HD-GYP domain protein ECA3548 from *Pectobacterium atrosepticum* lacks apparent c-di-GMP phosphodiesterase activity (and lacks the conserved HD motif), yet still impacts c-di-GMP regulated processes, possibly via direct interactions with other proteins [68]. The HD-GYP domain proteins of *V. cholerae* may also rely on protein-protein interactions to mediate their effects, and the necessary interactions may not have been achieved through over-expression or mutagenesis.

Finally, there is potential for phenotypic redundancy among the HD-GYP domain phosphodiesterases, as well as with EAL domain phosphodiesterases. Upon mutation of each of the GGDEF and EAL domain genes in *V. cholerae*, and only some mutants showed altered motility and/or biofilm development [41,45]. Therefore the chance of observing an effect of mutating the HD-GYP subset of c-di-GMP phosphodiesterases may be low. This is supported by our finding that individually, mutation of HD-GYP genes had no effect on the ability of *V. cholerae* to colonize the infant mouse small intestine, but a combination of mutations (ΔHDGYP7) significantly impaired colonization. It is possible that a combination of two or more HD-GYP domain proteins, or that the additive effects of multiple c-di-GMP PDEs, influence virulence of *V. cholerae*. These findings suggest that HD-GYP domain proteins as a family influence c-di-GMP levels during infection, which has previously been shown to impact *V. cholerae* virulence by regulating the virulence gene regulator ToxT [14,15].

In the context of an infection, discrete extracellular signals may trigger the production or activity of a specific c-di-GMP phosphodiesterase (or diguanylate cyclase), with cumulative but targeted effects on virulence gene expression. Strictly speaking, the c-di-GMP phosphodiesterases involved would not be mechanistically redundant (for example, having different interacting partners or subcellular localization), but work together toward the same phenotypic output. Such a regulatory system may allow *V. cholerae* to induce virulence gene expression in the host despite the absence or mitigation of one specific extracellular signal. A different set of c-di-GMP metabolic enzymes could modulate biofilm or motility in response to a distinct set of signals.

## Conclusions

Compared to GGDEF domain diguanylate cyclases and EAL domain phosphodiesterases, the HD-GYP domain phosphodiesterases have been understudied. This may be partly due to the fact that genes encoding HD-GYP domains are less widespread in bacterial genomes than those encoding GGDEF and EAL domains [38,65]. In *V. cholerae*, only a subset of the HD-GYP domain genes encoded are functional c-di-GMP PDEs. The individual HD-GYP domain genes are dispensable for motility and biofilm formation under the conditions tested, but collectively two or more of the genes are important for virulence of *V. cholerae*. Additional studies of HD-GYP domain proteins from other bacteria are needed to gain a full understanding their roles in c-di-GMP signaling.

## Additional file

Additional file 1: Table S1. Strains used in this study. **Table S2.** Plasmids used in this study. **Table S3.** Primers used in this study.

## References

[1] Ross P, Weinhouse H, Aloni Y, Michaeli D, Weinberger-Ohana P, Mayer R, Braun S, de Vroom E, van der Marel GA, van Boom JH, Benziman M (1987). Regulation of cellulose synthesis in *Acetobacter xylinum* by cyclic diguanylic acid. Nature.

[2] Hengge R (2009). Principles of c-di-GMP signalling in bacteria. Nat Rev Microbiol.

[3] Tamayo R, Pratt JT, Camilli A (2007). Roles of cyclic diguanylate in the regulation of bacterial pathogenesis. Annu Rev Microbiol.

[4] Cotter PA, Stibitz S (2007). c-di-GMP-mediated regulation of virulence and biofilm formation. Curr Opin Microbiol.

[5] Ahmad I, Lamprokostopoulou A, Le Guyon S, Streck E, Barthel M, Peters V, Hardt WD, Romling U (2011). Complex c-di-GMP signaling networks mediate transition between virulence properties and biofilm formation in *Salmonella enterica* serovar Typhimurium. PLoS One.

[6] Allombert J, Lazzaroni JC, Bailo N, Gilbert C, Charpentier X, Doublet P, Vianney A (2014). Three antagonistic cyclic di-GMP-catabolizing enzymes promote differential Dot/Icm effector delivery and intracellular survival at the early steps of *Legionella pneumophila* infection. Infect Immun.

[7] Bobrov AG, Kirillina O, Ryjenkov DA, Waters CM, Price PA, Fetherston JD, Mack D, Goldman WE, Gomelsky M, Perry RD (2011). Systematic analysis of cyclic di-GMP signalling enzymes and their role in biofilm formation and virulence in *Yersinia pestis*. Mol Microbiol.

[8] Huang CJ, Wang ZC, Huang HY, Huang HD, Peng HL (2013). YjcC, a c-di-GMP phosphodiesterase protein, regulates the oxidative stress response and virulence of *Klebsiella pneumoniae* CG43. PLoS One.

[9] Kulasakara H, Lee V, Brencic A, Liberati N, Urbach J, Miyata S, Lee DG, Neely AN, Hyodo M, Hayakawa Y, Ausubel FM, Lory S (2006). Analysis of *Pseudomonas aeruginosa* diguanylate cyclases and phosphodiesterases reveals a role for bis-(3′-5′)-cyclic-GMP in virulence. Proc Natl Acad Sci U S A.

[10] Lamprokostopoulou A, Monteiro C, Rhen M, Romling U (2010). Cyclic di-GMP signalling controls virulence properties of *Salmonella enterica* serovar typhimurium at the mucosal lining. Environ Microbiol.

[11] Lee HS, Gu F, Ching SM, Lam Y, Chua KL (2010). CdpA is a *Burkholderia pseudomallei* cyclic di-GMP phosphodiesterase involved in autoaggregation, flagellum synthesis, motility, biofilm formation, cell invasion, and cytotoxicity. Infect Immun.

[12] McKee RW, Mangalea MR, Purcell EB, Borchardt EK, Tamayo R (2013). The second messenger cyclic Di-GMP regulates *Clostridium difficile* toxin production by controlling expression of *sigD*. J Bacteriol.

[13] Sultan SZ, Pitzer JE, Miller MR, Motaleb MA (2010). Analysis of a *Borrelia burgdorferi* phosphodiesterase demonstrates a role for cyclic-di-guanosine monophosphate in motility and virulence. Mol Microbiol.

[14] Tamayo R, Schild S, Pratt JT, Camilli A (2008). Role of cyclic Di-GMP during El Tor biotype *Vibrio cholerae* infection: Characterization of the *in vivo*-induced cyclic-di-GMP phosphodiesterase CdpA. Infect Immun.

[15] Tischler AD, Camilli A (2005). Cyclic diguanylate regulates *Vibrio cholerae* virulence gene expression. Infect Immun.

[16] Zheng Y, Sambou T, Bogomolnaya LM, Cirillo JD, McClelland M, Andrews-Polymenis H (2013). The EAL domain containing protein STM2215 (rtn) is needed during *Salmonella* infection and has cyclic di-GMP phosphodiesterase activity. Mol Microbiol.

[17] Zogaj X, Wyatt GC, Klose KE (2012). Cyclic di-GMP stimulates biofilm formation and inhibits virulence of *Francisella novicida*. Infect Immun.

[18] Ausmees N, Mayer R, Weinhouse H, Volman G, Amikam D, Benziman M, Lindberg M (2001). Genetic data indicate that proteins containing the GGDEF domain possess diguanylate cyclase activity. FEMS Microbiol Lett.

[19] Chang AL, Tuckerman JR, Gonzalez G, Mayer R, Weinhouse H, Volman G, Amikam D, Benziman M, Gilles-Gonzalez MA (2001). Phosphodiesterase A1, a regulator of cellulose synthesis in *Acetobacter xylinum*, is a heme-based sensor. Biochemistry.

[20] Christen M, Christen B, Folcher M, Schauerte A, Jenal U (2005). Identification and characterization of a cyclic di-GMP specific phosphodiesterase and its allosteric control by GTP. J Biol Chem.

[21] Ryan RP, Fouhy Y, Lucey JF, Crossman LC, Spiro S, He YW, Zhang LH, Heeb S, Camara M, Williams P, Dow JM (2006). Cell-cell signaling in *Xanthomonas campestris* involves an HD-GYP domain protein that functions in cyclic di-GMP turnover. Proc Natl Acad Sci U S A.

[22] Ryjenkov DA, Tarutina M, Moskvin OV, Gomelsky M (2005). Cyclic diguanylate is a ubiquitous signaling molecule in bacteria: insights into biochemistry of the GGDEF protein domain. J Bacteriol.

[23] Schmidt AJ, Ryjenkov DA, Gomelsky M (2005). The ubiquitous protein domain EAL is a cyclic diguanylate-specific phosphodiesterase: enzymatically active and inactive EAL domains. J Bacteriol.

[24] Tamayo R, Tischler AD, Camilli A (2005). The EAL domain protein VieA is a cyclic diguanylate phosphodiesterase. J Biol Chem.

[25] Slater H, Alvarez-Morales A, Barber CE, Daniels MJ, Dow JM (2000). A two-component system involving an HD-GYP domain protein links cell-cell signalling to pathogenicity gene expression in *Xanthomonas campestris*. Mol Microbiol.

[26] Dow JM, Crossman L, Findlay K, He YQ, Feng JX, Tang JL (2003). Biofilm dispersal in *Xanthomonas campestris* is controlled by cell-cell signaling and is required for full virulence to plants. Proc Natl Acad Sci U S A.

[27] Chin KH, Lee YC, Tu ZL, Chen CH, Tseng YH, Yang JM, Ryan RP, McCarthy Y, Dow JM, Wang AH, Chou SH (2010). The cAMP receptor-like protein CLP is a novel c-di-GMP receptor linking cell-cell signaling to virulence gene expression in *Xanthomonas campestris*. J Mol Biol.

[28] He YW, Ng AY, Xu M, Lin K, Wang LH, Dong YH, Zhang LH (2007). *Xanthomonas campestris* cell-cell communication involves a putative nucleotide receptor protein Clp and a hierarchical signalling network. Mol Microbiol.

[29] Leduc JL, Roberts GP (2009). Cyclic di-GMP allosterically inhibits the CRP-like protein (Clp) of *Xanthomonas axonopodis* pv. citri. J Bacteriol.

[30] Tao F, He YW, Wu DH, Swarup S, Zhang LH (2010). The cyclic nucleotide monophosphate domain of *Xanthomonas campestris* global regulator Clp defines a new class of cyclic di-GMP effectors. J Bacteriol.

[31] Dow JM, Fouhy Y, Lucey JF, Ryan RP (2006). The HD-GYP domain, cyclic di-GMP signaling, and bacterial virulence to plants. Mol Plant Microbe Interact.

[32] Zhang Y, Wei C, Jiang W, Wang L, Li C, Wang Y, Dow JM, Sun W (2013). The HD-GYP domain protein RpfG of *Xanthomonas oryzae* pv. Oryzicola regulates synthesis of extracellular polysaccharides that contribute to biofilm formation and virulence on rice. PLoS One.

[33] Ryan RP, Lucey J, O’Donovan K, McCarthy Y, Yang L, Tolker-Nielsen T, Dow JM (2009). HD-GYP domain proteins regulate biofilm formation and virulence in *Pseudomonas aeruginosa*. Environ Microbiol.

[34] Ryan RP (2013). Cyclic di-GMP signalling and the regulation of bacterial virulence. Microbiology.

[35] Stelitano V, Giardina G, Paiardini A, Castiglione N, Cutruzzola F, Rinaldo S (2013). C-di-GMP hydrolysis by *pseudomonas aeruginosa* HD-GYP phosphodiesterases: Analysis of the reaction mechanism and novel roles for pGpG. PLoS One.

[36] Sultan SZ, Pitzer JE, Boquoi T, Hobbs G, Miller MR, Motaleb MA (2011). Analysis of the HD-GYP domain cyclic dimeric GMP phosphodiesterase reveals a role in motility and the enzootic life cycle of *Borrelia burgdorferi*. Infect Immun.

[37] Galperin MY, Nikolskaya AN, Koonin EV (2001). Novel domains of the prokaryotic two-component signal transduction systems. FEMS Microbiol Lett.

[38] Galperin MY, Higdon R, Kolker E (2010). Interplay of heritage and habitat in the distribution of bacterial signal transduction systems. Mol Biosyst.

[39] Rashid MH, Rajanna C, Ali A, Karaolis DK (2003). Identification of genes involved in the switch between the smooth and rugose phenotypes of *Vibrio cholerae*. FEMS Microbiol Lett.

[40] Tischler AD, Camilli A (2004). Cyclic diguanylate (c-di-GMP) regulates *Vibrio cholerae* biofilm formation. Mol Microbiol.

[41] Liu X, Beyhan S, Lim B, Linington RG, Yildiz FH (2010). Identification and characterization of a phosphodiesterase that inversely regulates motility and biofilm formation in *Vibrio cholerae*. J Bacteriol.

[42] Lim B, Beyhan S, Yildiz FH (2007). Regulation of vibrio polysaccharide synthesis and virulence factor production by CdgC, a GGDEF-EAL domain protein, in *Vibrio cholerae*. J Bacteriol.

[43] Lim B, Beyhan S, Meir J, Yildiz FH (2006). Cyclic-diGMP signal transduction systems in *Vibrio cholerae*: Modulation of rugosity and biofilm formation. Mol Microbiol.

[44] Beyhan S, Yildiz FH (2007). Smooth to rugose phase variation in *Vibrio cholerae* can be mediated by a single nucleotide change that targets c-di-GMP signalling pathway. Mol Microbiol.

[45] Beyhan S, Odell LS, Yildiz FH (2008). Identification and characterization of cyclic diguanylate signaling systems controlling rugosity in *Vibrio cholerae*. J Bacteriol.

[46] Pratt JT, McDonough E, Camilli A (2009). PhoB regulates motility, biofilms, and cyclic di-GMP in *Vibrio cholerae*. J Bacteriol.

[47] Hammer BK, Bassler BL (2009). Distinct sensory pathways in *Vibrio cholerae* El Tor and classical biotypes modulate cyclic dimeric GMP levels to control biofilm formation. J Bacteriol.

[48] Koestler BJ, Waters CM (2014). Bile acids regulate intracellular cyclic di-GMP in *Vibrio cholerae*. Infect Immun.

[49] Donnenberg MS, Kaper JB (1991). Construction of an *eae* deletion mutant of enteropathogenic *Escherichia coli* by using a positive-selection suicide vector. Infect Immun.

[50] Hickman JW, Tifrea DF, Harwood CS (2005). A chemosensory system that regulates biofilm formation through modulation of cyclic diguanylate levels. Proc Natl Acad Sci U S A.

[51] Ross P, Aloni Y, Weinhouse H, Michaeli D, Weinberger-Ohana P, Mayer R, Benziman M (1986). Control of cellulose biosynthesis in *Acetobacter xylinum*. A unique guanyl oligonucleotide is the immediate activator of the cellulose synthase. Carbohydr Res.

[52] Lauriano CM, Ghosh C, Correa NE, Klose KE (2004). The sodium-driven flagellar motor controls exopolysaccharide expression in *Vibrio cholerae*. J Bacteriol.

[53] Tamayo R, Patimalla B, Camilli A (2010). Growth in a biofilm induces a hyperinfectious phenotype in *Vibrio cholerae*. Infect Immun.

[54] Pratt JT, Tamayo R, Tischler AD, Camilli A (2007). PilZ domain proteins bind cyclic diguanylate and regulate diverse processes in *Vibrio cholerae*. J Biol Chem.

[55] Sambrook J, Fritsch EF, Maniatis T (1989). Molecular Cloning: a Laboratory Manual.

[56] Mudrak B, Tamayo R (2012). The *Vibrio cholerae* Pst2 phosphate transport system is upregulated in biofilms and contributes to biofilm-induced hyperinfectivity. Infect Immun.

[57] Airola MV, Sukomon N, Samanta D, Borbat PP, Freed JH, Watts KJ, Crane BR (2013). HAMP domain conformers that propagate opposite signals in bacterial chemoreceptors. PLoS Biol.

[58] Aravind L, Ponting CP (1999). The cytoplasmic helical linker domain of receptor histidine kinase and methyl-accepting proteins is common to many prokaryotic signalling proteins. FEMS Microbiol Lett.

[59] Williams SB, Stewart V (1999). Functional similarities among two-component sensors and methyl-accepting chemotaxis proteins suggest a role for linker region amphipathic helices in transmembrane signal transduction. Mol Microbiol.

[60] Miner KD, Klose KE, Kurtz DM (2013). An HD-GYP cyclic di-guanosine monophosphate phosphodiesterase with a non-heme diiron-carboxylate active site. Biochemistry.

[61] Bellini D, Caly DL, McCarthy Y, Bumann M, An SQ, Dow JM, Ryan RP, Walsh MA (2014). Crystal structure of an HD-GYP domain cyclic-di-GMP phosphodiesterase reveals an enzyme with a novel trinuclear catalytic iron centre. Mol Microbiol.

[62] Lovering AL, Capeness MJ, Lambert C, Hobley L, Sockett RE: **The structure of an unconventional HD-GYP protein from*****Bdellovibrio*****reveals the roles of conserved residues in this class of cyclic-di-GMP phosphodiesterases.** *MBio* 2011, **2**(5), doi: 10.1128/mBio.00163-11. 10.1128/mBio.00163-11 PMC3188283 21990613

[63] Beyhan S, Tischler AD, Camilli A, Yildiz FH (2006). Transcriptome and phenotypic responses of *Vibrio cholerae* to increased cyclic di-GMP level. J Bacteriol.

[64] Casper-Lindley C, Yildiz FH (2004). VpsT is a transcriptional regulator required for expression of *vps* biosynthesis genes and the development of rugose colonial morphology in *Vibrio cholerae* O1 El Tor. J Bacteriol.

[65] Romling U, Galperin MY, Gomelsky M (2013). Cyclic di-GMP: the first 25 years of a universal bacterial second messenger. Microbiol Mol Biol Rev.

[66] Ryan RP, Dow JM (2010). Intermolecular interactions between HD-GYP and GGDEF domain proteins mediate virulence-related signal transduction in *Xanthomonas campestris*. Virulence.

[67] Andrade MO, Alegria MC, Guzzo CR, Docena C, Rosa MC, Ramos CH, Farah CS (2006). The HD-GYP domain of RpfG mediates a direct linkage between the Rpf quorum-sensing pathway and a subset of diguanylate cyclase proteins in the phytopathogen *Xanthomonas axonopodis* pv citri. Mol Microbiol.

[68] Tan H, West JA, Ramsay JP, Monson RE, Griffin JL, Toth IK, Salmond GP (2014). Comprehensive overexpression analysis of cyclic-di-GMP signalling proteins in the phytopathogen *Pectobacterium atrosepticum* reveals diverse effects on motility and virulence phenotypes. Microbiology.

