# Supplementary material for: A systematic analysis of the in vitro and in vivo functions of the HD-GYP domain proteins of Vibrio cholerae
Source: BMC Microbiol. 2014 Oct 25;14:272. doi: 10.1186/s12866-014-0272-9 (PMC4212101; doi:10.1186/s12866-014-0272-9)
Supplement: Additional file 1: Table S1. — Strains used in this study. Table S2. Plasmids used in this study. Table S3. Primers used in this study. [file 12866_2014_272_MOESM1_ESM.pdf]

SUPPLEMENTAL TABLES S1-S3.

A systematic analysis of the *in vitro* and *in vivo* functions of the HD-GYP domain proteins of *Vibrio cholerae*

Robert W. McKee, Ankunda Kariisa, Benjamin Mudrak, Courtney Whitaker and Rita Tamayo*

Department of Microbiology and Immunology, University of North Carolina at Chapel Hill, Chapel Hill, NC, USA

*Corresponding Author Contact Information

Department of Microbiology and Immunology

University of North Carolina at Chapel Hill

125 Mason Farm Rd, CB# 7290

(919) 843-2864

rtamayo@med.unc.edu

Table S1. Strains used in this study

Strain	Description	Reference
V. cholerae		
	C6706, O1 El Tor Inaba	[1]
	N16961, O1 El Tor Inaba	[2]
	C6706 Δ*vpsR*	[3]
	C6706 *flaA*::pGP704	This work
	C6706 Δ*lacZ*	[4]
	C6706 ΔVC1087	This work
	C6706 ΔVC1295	This work
	C6706 ΔVC1348	This work
	C6706 ΔVC2340	This work
	C6706 VC2497::pGP704	This work
	C6706 ΔVCA0210	This work
	C6706 ΔVCA0681	This work
	C6706 ΔVCA0895	This work
	C6706 ΔVCA0931	This work
	C6706 ΔVCA0681 ΔVCA0210 ΔVC2340 ΔVC1348 ΔVCA0895 ΔVC1295 ΔVCA0931 "ΔHDGYP7"	This work
	C6706 pMMB67EH	This work
	C6706 pMMB67EH::VC1592-His6	This work
	C6706 pMMB67EH::VC1087-His6	This work
	C6706 pMMB67EH::VC1295-His6	This work
	C6706 pMMB67EH::VC1348-His6	This work
	C6706 pMMB67EH::VC1348AA-His6	This work
	C6706 pMMB67EH::VC2340-His6	This work
	C6706 pMMB67EH::VC2497-His6	This work
	C6706 pMMB67EH::VCA0210-His6	This work
	C6706 pMMB67EH::VCA0681-His6	This work
	C6706 pMMB67EH::VCA0895-His6	This work
	C6706 pMMB67EH::VCA0931-His6	This work
	C6706 pMMB67EH::VCA0895(HD-GYP)-His6	This work
E. coli		
	DH5α; F^- Δ(*lacZYA-argF*)*U169 recA1 endA1 hsdR17 supE44 thi-1 gvrA96 relA1*	Invitrogen, [5]
	DH5α*λpir;* F^- Δ(*lacZYA-argF*)*U169 recA1 endA1 hsdR17 supE44 thi-1 gvrA96 relA1 λpir*	[6]
	SM10*λpir; thi recA thr leu tonA lacY supE* RP4-2 Tc::Mu *λpir*	[7]
	E. coli (pRK2013::Tn9). KnR, CmR. pRK2013::Tn9 has IncP1tra and oriColE1.	[8]
	BL21; *fhuA2 [lon] ompT gal [dcm] ΔhsdS*	New England Biolabs
	BL21 pMMB67EH	This work
	BL21 pMMB67EH::VC1087-His6	This work
	BL21pMMB67EH::VC1295-His6	This work
	BL21 pMMB67EH::VC1348-His6	This work
	BL21 pMMB67EH::VC2340-His6	This work

	BL21 pMMB67EH::VC2497-His6	This work
	BL21 pMMB67EH::VCA0210-His6	This work
	BL21 pMMB67EH::VCA0681-His6	This work
	BL21 pMMB67EH::VCA0895-His6	This work
	BL21 pMMB67EH::VCA0931-His6	This work
	BL21 pMMB67EH::VCA0895(HD-GYP)-His6	This work

Table S2. Plasmids used in this study

Plasmid	Description	Reference
pCVD442	Suicide vector for allelic replacement using *sacB* counterselection; *oriR6K mobRP4 sacB*, Ap^r	[9]
pCVD442::ΔVC1087	Suicide vector for deleting VC1087	This work
pCVD442::ΔVC1295	Suicide vector for deleting VC1295	This work
pCVD442::ΔVC1348	Suicide vector for deleting VC1348	This work
pCVD442::ΔVC2340	Suicide vector for deleting VC2340	This work
pCVD442::ΔVC2497	Suicide vector for deleting VC2497	This work
pCVD442::ΔVCA0210	Suicide vector for deleting VCA0210	This work
pCVD442::ΔVCA0681	Suicide vector for deleting VCA0681	This work
pCVD442::ΔVCA0895	Suicide vector for deleting VCA0895	This work
pCVD442::ΔVCA0931	Suicide vector for deleting VCA0931	This work
pGP704	Suicide vector; *oriR6K mobRP4*, Ap^r	[7]
pGP704::'*flaA*'	Suicide vector for pGP704 insertion in *flaA*	[10]
pGP704::'VC2497'	Suicide vector for pGP704 insertion in VC2497	This work
pMMB67EH	IncQ broad-host-range low-copy-number cloning vector, IPTG inducible, Ap^r	[11]
pMMB67EH::VC1087-His6	Expression vector for VC1087	This work
pMMB67EH::VC1295-His6	Expression vector for VC1295 from N16961	This work
pMMB67EH::VC1348-His6	Expression vector for VC1348	This work
pMMB67EH::VC1348AA-His6	Expression vector for VC1348 with alanine substitutions in the HD motif	This work
pMMB67EH::VC2340-His6	Expression vector for VC2340	This work
pMMB67EH::VC2497-His6	Expression vector for VC2497	This work
pMMB67EH::VCA0210-His6	Expression vector for VCA0210	This work
pMMB67EH::VCA0681-His6	Expression vector for VCA0681	This work
pMMB67EH::VCA0895-His6	Expression vector for VCA0895	This work
pMMB67EH::VCA0931-His6	Expression vector for VCA0931	This work
pMMB67EH::VCA0895(HDGYP)-His6	Expression vector for HD-GYP domain from VCA0895	This work

Table S3. Primers used in this study

Primer Name	Primer Sequence (5’ to 3’)
RPB2F	CTGTCTCAAGCCGGTTACAA[a]
RPB2R	TTTCTACCAGTGCAGAGATGC [a]
1087F1	CAGAGCTCTCCCTATCTTTGGGTTGG
1087R1	GGCCATGGATCGTGCATGTTATCCATTTG
1087F2	GGCCATGGTAAGGACACAGGATAAGGATTC
1087R2	GGGCATGCAAGCTGGTTGAAACCGAG
1087F0	GGTGGATTGCCGATATTG
1295F1	GAGCATGCGCCAAGACGGGAATATCG
1295R1	GGCCATGGGGTCAATAAGTTACGGCTAGT
1295F2	GGCCATGGGTGGTCGAGGCTTTTCTCG
1295R2	CCGAGCTCCAGATTTAGAGCAGGCAGAG
1295F0	AGATCATCACTGGGTCGAG
1348F1	CCGAGCTCCGCTCGCACTTCAGATTC
1348R1	CACCATGGGGTCTTCCATTGACATGTG
1348F2	CACCATGGACCCTATGGTGATTGATGCC
1348R2	GTGCATGCTGGTTATTGGCGTTCGTC
1348F0	CGGATGGTTGCGAATGATG
2340F1	CCGCATGCCTTGGCACCACAACAGTC
2340R1	GGCCATGGATATCACCGCCCTTCCTTG
2340F2	CACCATGGACAAGATCCCAAGGACCAG
2340R2	CACGAGCTCCTCTTCACTGGGTTGATGC
2340F0	GCAGCCATCTCCTTGATAC
A0210F1	GGTCTAGAGTGTATGATACCGCAATCG
A0210R1	CACCATGGCCACTTCAACCTTCTTTAGG
A0210F2	GGCCATGGGACTAGGACAGCGACAAAAG
A0210R2	CCGAGCTCCTGTCACCGCAATACCTAAC
A0210F0	GTACGCGCATTTCTTCACG
A0681F1	CAGCATGCCATCCACGTTATTGGTTAGG
A0681R1	GGCCATGGGACCATCTCATTGCTCATCC
A0681F2	GACCATGGGGAAAGCCCAACCGAATG
A0681R2	CCGAGCTCCTATCGAGCCCAAGTAAAGC
A0681F0	GGCGAGCTAACAAATAGACC
A0895F1	GGGCATGCTGCCAGTGGTGATGTAGG
A0895R1	GGCCATGGGCTAGGTTGCAAAAAATCACG
A0895F2	GGCCATGGTAGAACATCACGACAGAAAGG
A0895R2	CAGAGCTCGCAGATTGCAGATCGTAACC
A0895F0	AACTCTTCAATCGGCTGG
A0931F1	CCGAGCTCCGAGATGTTCTGCGAGAC
A0931R1	GGCGTCGACAGGGAAGGTATTTTGTGCAAC
A0931F2	CACGTCGACCAGATAAGACCTAACAAGGC
A0931R2	CGGCATGCTTTGTGTGAGCATCGAGTG
A0931F0	AAGGAATACACGGGCAAC
2497koF	GTGAGATCTGGCTCTACATTCGGTTGC
2497koR	GAGAATTCGTTTAGCACTTTCAGGAAACG
1087qF	CATCAGCAAACCTTGGGACA
1087qR	CAGCTAATGCCTGATTCGCA

1295qF	CTTTACACAAGCCGAGTCTGTC
1295qR	GAAGCAGAGTATGCCGAATCAG
1348qF	ATCGCCTTTATGTCGCAAGG
1348qR	TGACCTCATAGCCCGACATT
2340qF	GCACTGCGCATTATTCACGA
2340qR	AAACCGACGAAGGTGTGTTG
2497qF	CAAGCGTTGAAGAGGCAACT
2497qR	CGACGTCGATATTCGTTGAGC
A0210qF	AAGAGCCTTGGTGTGTGTTG
A0210qR	TCTAGGCCAGAAAGAACCGA
A0681qF	AGAGGAGTGTCAACTGGTGT
A0681qR	AATGCTGCTGGGTATTGTCG
A0895qF	AGCTCGAAACCGTGTTTACC
A0895qR	GTTGATCTTGTAGTGGCGGTTC
A0931qF	TACTGAAAGATGCGGGTTGC
A0931qR	ACTGGAAAGACTGGTGCCTA
VC1087F	GGGAGCTCTTTAGGATACATTTTT**ATG**CAAATGGATAACATGCAC
VC1087HR	AATCTAGACTA*ATGGTGATGGTGATGGTG*TCCTATATAAACGGGGATCGG
VC1295F	CGTCTAGATTTAGGATACATTTTT**TTG**ACCATTTGGGTTCTATCAC
VC1295HR	TTGCATGCCTA*ATGGTGATGGTGATGGTG*TGCGGCATCTTTAAAGTGT
VC1348F	GGGAGCTCTTTAGGATACATTTTT**ATG**GCAACCGCCAATATC
VC1348HR	TATCTAGACTA*ATGGTGATGGTGATGGTG*GCCTGACGCTTGTTGAC
VC1348R1-AA	CTTTACCTATGGCGGCCAACG
VC1348F2-AA	CGTTGCACGACATAGGTAAAG
VC2340F	GGTCTAGATTTAGGATACATTTTT**ATG**CAGATGCAACCCA
VC2340HR	TTGCATGCCTA*ATGGTGATGGTGATGGTG*GTGGGTGATTCCCTGG
VC2497F	GGGAGCTCTTTAGGATACATTTTT**GTG**GCAAGCATTAAAATCACG
VC2497HR	TAGCATGCCTA*ATGGTGATGGTGATGGTG*CTCTTCGCTGTCAAAGAAGT
VCA0210F	GGGAGCTCTTTAGGATACATTTTT**TTG**AAGTGGTTTAAATATGGAGATG
VCA0210HR	TATCTAGACTA*ATGGTGATGGTGATGGTG*GTCAGGCAGCGAAGC
VCA0681F	GGTCTAGATTTAGGATACATTTTT**ATG**AGATGGTCAGAAATAGGC
VCA0681HR	TAGCATGCCTA*ATGGTGATGGTGATGGTG*TGCCCATTCGGTTGG
VCA0895F	GGGAGCTCTTTAGGATACATTTTT**TTG**CAACCTAGCCAAGAT
VCA0895HR	TATCTAGACTA*ATGGTGATGGTGATGGTG*GTAATCTTCCCGTAAATACGC
VCA0931F	GGGAGCTCTTTAGGATACATTTTT**ATG**AGTGTTGCACAAAATACC
VCA0931HR	TATCTAGACTA*ATGGTGATGGTGATGGTG*GGTCTTATCTGAAGAGTGTGG
A0895Fb	GG*GAGCTC*TTTAGGATACATTTTT**ATG**CGTTTATTAGCCGAAGCC

Underlined= restriction site sequence
Bolded= translational start site (native)
Italics= six-histidine sequence
[a] qRT-PCR primers to *rpoB* described previously [12]

References

1. Thelin KH, Taylor RK: **Toxin-coregulated pilus, but not mannose-sensitive hemagglutinin, is required for colonization by *Vibrio cholerae* O1 El Tor biotype and O139 strains.** *Infect Immun* 1996, **64**(7):2853-6.

2. Richardson K, Parker CD: **Identification and occurrence of *Vibrio cholerae* flagellar core proteins in isolated outer membrane.** *Infect Immun* 1985, **47**(3):674-9.

3. Mudrak B, Tamayo R: **The *Vibrio cholerae* Pst2 phosphate transport system is upregulated in biofilms and contributes to biofilm-induced hyperinfectivity.** *Infect Immun* 2012, **80**(5):1794-1802.

4. Tamayo R, Patimalla B, Camilli A: **Growth in a biofilm induces a hyperinfectious phenotype in *Vibrio cholerae*.** *Infect Immun* 2010, **78**(8):3560-3569.

5. Hanahan D: In *DNA Cloning: A Practical Approach. Volume 1.* Edited by Glover DM. McLean, VA: IRL Press; 1985:109.

6. Elliott SJ, Kaper JB: **Role of type 1 fimbriae in EPEC infections.** *Microb Pathog* 1997, **23**(2):113-118.

7. Miller VL, Mekalanos JJ: **A novel suicide vector and its use in construction of insertion mutations: osmoregulation of outer membrane proteins and virulence determinants in *Vibrio cholerae* requires *toxR*.** *J Bacteriol* 1988, **170**(6):2575-83.

8. Ditta G, Stanfield S, Corbin D, Helinski DR: **Broad host range DNA cloning system for gram-negative bacteria: construction of a gene bank of Rhizobium meliloti.** *Proc Natl Acad Sci U S A* 1980, **77**(12):7347-51.

9. Donnenberg MS, Kaper JB: **Construction of an *eae* deletion mutant of enteropathogenic *Escherichia coli* by using a positive-selection suicide vector.** *Infect Immun* 1991, **59**(12):4310-7.

10. Moisi M, Jenul C, Butler SM, New A, Tutz S, Reidl J, Klose KE, Camilli A, Schild S: **A novel regulatory protein involved in motility of *Vibrio cholerae*.** *J Bacteriol* 2009, **191**(22):7027-7038.

11. Morales VM, Backman A, Bagdasarian M: **A series of wide-host-range low-copy-number vectors that allow direct screening for recombinants.** *Gene* 1991, **97**(1):39-47.

12. Quinones M, Kimsey HH, Waldor MK: **LexA cleavage is required for CTX prophage induction.** *Mol Cell* 2005, **17**(2):291-300.
